# Efficient inference in state-space models through adaptive learning in online Monte Carlo expectation maximization

**DOI:** 10.1007/s00180-019-00937-4

**Published:** 2019-12-03

**Authors:** Donna Henderson, Gerton Lunter

**Affiliations:** 1 Wellcome Centre of Human Genetics, University of Oxford, Oxford, OX3 7BN UK; 2 MRC Weatherall Institute of Molecular Medicine, Unversity of Oxford, Oxford, OX3 9DS UK

**Keywords:** Stochastic approximation expectation maximization, Sequential Monte Carlo, Latent variable model, Online estimation

## Abstract

Expectation maximization (EM) is a technique for estimating maximum-likelihood parameters of a latent variable model given observed data by alternating between taking expectations of sufficient statistics, and maximizing the expected log likelihood. For situations where sufficient statistics are intractable, stochastic approximation EM (SAEM) is often used, which uses Monte Carlo techniques to approximate the expected log likelihood. Two common implementations of SAEM, Batch EM (BEM) and online EM (OEM), are parameterized by a “learning rate”, and their efficiency depend strongly on this parameter. We propose an extension to the OEM algorithm, termed Introspective Online Expectation Maximization (IOEM), which removes the need for specifying this parameter by adapting the learning rate to trends in the parameter updates. We show that our algorithm matches the efficiency of the optimal BEM and OEM algorithms in multiple models, and that the efficiency of IOEM can exceed that of BEM/OEM methods with optimal learning rates when the model has many parameters. Finally we use IOEM to fit two models to a financial time series. A Python implementation is available at https://github.com/luntergroup/IOEM.git.

## Introduction

Expectation Maximization (EM) is a general and widely used technique for estimating maximum likelihood parameters of latent variable models (Dempster et al. 1977). It involves iterating two steps: computing the expected log-likelihood marginalizing over the latent variable conditioned on parameters and data (the E step), and optimizing parameters to maximize this expected log-likelihood (the M step). In important special cases the E-step is analytically tractable; examples include linear systems with Gaussian noise (Shumway and Stoffer 1982) and finite-state hidden Markov models (Baum 1972). In general however, Monte Carlo techniques such as Stochastic EM (SEM; Celeux and Diebolt 1985; Celeux et al. 1995) and Monte Carlo EM (MCEM; Wei and Tanner 1990) are necessary to approximate the required integral. The stochastic nature of Monte Carlo techniques result in noisy parameter estimates, and to address this, methods such as Stochastic Approximation EM (SAEM; Nowlan 1991; Celeux and Diebolt 1992; Delyon et al. 1999) were developed that make smaller incremental updates parameterized by a learning rate $\gamma$ or learning schedule $\{\gamma _t\}$.

In this paper we focus on models where the latent variable has a longitudinal structure and follows a Markov model (see e.g. Lopes and Tsay 2010 for examples in financial econometrics). For such models, the required samples from the posterior distribution can be generated using Sequential Monte Carlo (SMC) techniques (see Doucet et al. 2001; Doucet and Johansen 2009 and references therein). In one approach, the Batch EM (BEM) algorithm processes a contiguous chunk of data to generate latent variable samples from the posterior, which are used in the M step to update parameters. An alternative approach is online EM (OEM; Mongillo and Denève 2008; Cappé 2009), in which parameters are continuously updated as data are processed. Analogous to SAEM, OEM algorithms have a parameter $\gamma$ controlling the learning rate, an idea apparently first introduced in this context by Jordan and Jacobs (1993). Several recent papers have addressed related problems. For instance Yildirim et al. (2013) use a particle filter to implement an online EM algorithm for change point models (see also Fearnhead 2006; Fearnhead and Vasileiou 2009), which uses a pre-specified learning schedule (called “step-size sequence” in their work) to control convergence. Le Corff and Fort (2013) introduced a “block online” EM algorithm for hidden Markov models that combines online and batch ideas, controlling convergence through a block size sequence $\tau _k$.

All these algorithms thus require choosing tuning parameters in the form of a batch size, block sequence, learning rate or a learning schedule. It turns out that this choice can strongly influences the performance of these algorithms. For instance, for BEM, very large batch sizes lead to inaccurate estimates because of slow convergence, whereas very small batch sizes lead to imprecise estimates due to the inherent stochasticity of the model within a small batch of observations. The optimal batch size in BEM or the optimal learning rate in OEM depends on the particularities of the model.

This raises the question of how to choose this tuning parameter. Several authors have proposed adaptive acceleration techniques for EM methods that obviate the need for choosing tuning parameters (Jamshidian and Jennrich 1993; Lange 1995; Varadhan and Roland 2008), but these methods require that the E-step is analytically tractable. In the context of (stochastic) gradient descent optimization (Bottou 2012), several influential adaptive algorithms have recently been proposed (Zeiler 2012; Kingma and Ba 2015; Mandt et al. 2016; Reddi et al. 2018) that have few or no tuning parameters. In principle, these methods can be used to find maximum likelihood parameters, but unless data is processed in batches, applying these methods to state-space models with a sequential structure is not straightforward. In addition, EM approaches enjoy several advantages over gradient descent methods, including automatic guarantees of parameter constraints and increased numerical stability (Xu and Jordan 1996; Cappé 2009; Kantas et al. 2009; Chitralekha et al. 2010).

Here we introduce a novel algorithm, termed Introspective Online EM (IOEM), which removes the need for setting the learning rate by estimating optimal parameter-specific learning rates from the data. This is particularly helpful when inferring parameters in a high dimensional model, since the optimal learning rate may differ between parameters. IOEM can be applied to inference in state-space models with observations $Y_t$ and state variables $X_t$ governed by transition probability function $f(x_{t+1}|x_t,\theta )$ and observation probability function $g(y_t|x_t,\theta )$, for which $f(x_{t}|x_{t-1},\theta )g(y_t|x_t,\theta )$ belongs to an exponential family with sufficient statistic $s(x_{t-1},x_t,y_t)$. Broadly, IOEM works by estimating both the precision and the accuracy of parameters in an online manner through weighted linear regression, and uses these estimates to tune the learning rate so as to improve both simultaneously.

The outline of this paper is as follows. Section 2 introduces BEM, OEM, and a simplified version of IOEM in the context of a one-parameter autoregressive state-space model. Section 3 introduces the complete IOEM algorithm required for inference in the full 3-parameter autoregressive model. Section 4 discusses simulation results of the algorithms for these two models. In addition we consider a 2-dimensional autoregressive model to show the benefit of the proposed algorithm when inferring many parameters, and we demonstrate desirable performance in the stochastic volatility model, an important case as it is nonlinear and hence relevant to actual applications of SAEM. In Sect. 5 we apply IOEM with the autoregressive and stochastic volatility models to a financial time series, and we end the paper with a brief discussion.

## EM algorithms for a simplified autoregressive model

Here we review BEM (Dempster et al. 1977), OEM (Cappé 2009) and SMC (Doucet and Johansen 2009), and present the IOEM algorithm in a simplified context. This illustrates the main ideas behind IOEM before presenting the full algorithm in Sect. 3.

We consider a simple noisily-observed autoregressive model with one unknown parameter, equivalent to an ARMA(1,1) model. We observe the sequence of random variables $Y_{1:t}:=\{Y_k\}_{k=1,\ldots ,t}$ that depend on the unobserved sequence $X_{1:t}:=\{X_k\}_{k=1,\ldots ,t}$ as follows:

1 $$\begin{aligned} X_t&=a X_{t-1}+ \sigma _w W_t, \nonumber \\ Y_t&=X_t + \sigma _v V_t, \end{aligned}$$

where $W_t$ and $V_t$ are i.i.d. standard normal variates, $a=0.95$ and $\sigma _w^2=1$ are known parameters, and $\sigma _v^2$ is unknown. Under this model, we have the following transition and emission densities:

$$\begin{aligned} f(x_t|x_{t-1})&= (2 \pi \sigma _w^2)^{-1/2} \exp \Big \{-\frac{(x_t - a x_{t-1})^2}{2 \sigma _w^2}\Big \}, \\ g(y_t|x_t)&= (2 \pi \sigma _v^2)^{-1/2} \exp \Big \{-\frac{(y_t- x_t)^2}{2 \sigma _v^2}\Big \}. \end{aligned}$$

We have chosen $\sigma _v^2$ as the unknown parameter as it is the most straightforward to estimate, allowing us to introduce the idea of IOEM while avoiding certain complications that we address in Sect. 3. As *f* and *g* are members of the exponential family of distributions, the M step of EM can be done using sufficient statistics, and the E step amounts to calculating their expectation. In this model, the parameter $\sigma _v^2$ has the sufficient statistic

2 $$\begin{aligned} S_t = \mathbb {E}_{X_{1:t} | Y_{1:t}, \theta }\left[ \frac{1}{t} \sum _{k=1}^{t}(Y_k-X_k)^2 \right] . \end{aligned}$$

The estimate of $\sigma _v^2$ is obtained by setting $\hat{\sigma }_{v,t}^2=\hat{S}_t$. More generally, for an unknown parameter $\theta$, $\hat{\theta }_t=\varLambda (\hat{S}_t)$ where $\varLambda$ is a known function mapping sufficient statistics to parameter estimates.

To estimate $S_t$, we use sequential Monte Carlo (SMC) to simulate particles $X^{(i)}_{1:t}$ and their associated weights $w(X^{(i)}_{1:t})$, $i=1,\ldots ,N$, so that

3 $$\begin{aligned} \sum _{i=1}^N w(X^{(i)}_{1:t}) \delta _{X^{(i)}_{1:t}} \end{aligned}$$

approximates the distribution $p(X_{1:t}|Y_{1:t},\theta )$. The standard MCEM approximation of $p(X_{1:t}|Y_{1:t},\hat{\theta })$ would require storage of all observations $Y_{1:t}$ and simulation of $X^{(i)}_{1:t}$ each time $\hat{\theta }$ is updated, and ideally an increasing Monte Carlo sample size as the parameter estimates near convergence. To avoid this, we employ SAEM (Celeux and Diebolt 1992) which effectively averages over previous parameter estimates as an alternative to generating a new Monte Carlo sample every time an estimate is updated, and hence is more suitable to online inference. This method as proposed in Cappé and Moulines (2009) approximates the expectation in (2) recursively.

The outline of the SMC with EM algorithm we consider in this paper is as follows (Doucet and Johansen 2009):
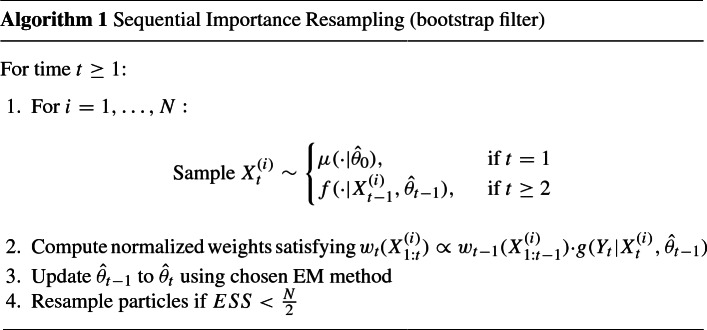


Here $\mu (\cdot | \hat{ \theta }_{0})$ is the initial distribution for $X_1$, *ESS* is the effective sample size defined as $[\sum _{i=1}^N w_t(X_{1:t}^{(i)})^{-2}]^{-1}$, $w_0(\cdot )=1/N$, and $X_{t}^{(i)}$ is shorthand for the $t^{\mathrm{th}}$ coordinate of $X_{1:t}^{(i)}$. In models with multiple unknown parameters, each parameter is updated in step 3 of the algorithm, however we will refer only to a single parameter $\theta$ to keep the notation simple.

Throughout this paper we follow common practice in using the fixed-lag technique in order to reduce the mean square error between $S_t$ and $\hat{S}_t$ (Cappé and Moulines 2005; Cappé et al. 2007). We choose a lag $\varDelta > 0$ and at time *t*, using particles $X_{1:t}^{(i)}$ shaped by data $Y_{1:t}$, we estimate the $t-\varDelta ^{\text {th}}$ term of the summation in (2). We will use $X_{1:t}^{(i)}(t-\varDelta )$ to denote the $t-\varDelta ^{\mathrm{th}}$ coordinate of the particle $X_{1:t}^{(i)}$, but we will continue to write $X_{t}^{(i)}$ as a shorthand for $X_{1:t}^{(i)}(t)$. (See Table 1 for an overview of notation used in this paper).

The fixed-lag technique involves making the approximation

$$\begin{aligned} S_t \approx \mathbb {E}_{X_{1:t}|Y_{1:t} , \theta }\left[ \frac{1}{t- \varDelta } \sum _{j=1}^{t-\varDelta } s(Y_j,X_j) \right] \approx \frac{1}{t- \varDelta } \sum _{j=1}^{t-\varDelta } \mathbb {E}_{X_{1:j+\varDelta }| Y_{1:j+\varDelta } , \hat{\theta } } \left[ s(Y_j,X_j) \right] \end{aligned}$$

where we assume that $S_t$ can be written as

$$\begin{aligned} S_t&= \mathbb {E}_{X_{1:t}|Y_{1:t},\theta } \sum _{j=1}^t s(Y_j,X_j) \end{aligned}$$

This allows $S_t$ to be updated in an online manner by computing the component-wise sufficient statistics

$$\begin{aligned} \tilde{s}_t&:= \mathbb {E}_{ X_{1:t}|Y_{1:t} , \theta } \left[ s( Y_{t-\varDelta }, X_{1:t}(t-\varDelta ) ) \right] \\&\approx \sum _i w_k(X_{1:t}^{(i)}) s(Y_{t-\varDelta }, X_{1:t}^{(i)}(t-\varDelta )), \end{aligned}$$

allowing $\hat{S}_t$ to be updated as

$$\begin{aligned} \hat{S}_t = \gamma _t \cdot \tilde{s}_t + (1-\gamma _t) \cdot \hat{S}_{t-1}, \end{aligned}$$

with some learning schedule $\gamma _t$; in (3) $\gamma _t=1/(t-\varDelta )$. This approach is slightly different from that of Cappé and Moulines (2005); see Sect. 7.1 for a discussion.

Choosing a large value of $\varDelta$ allows SMC to use many observations to improve the posterior distribution of $X_{t-\varDelta }$. However the cost of a large $\varDelta$ is an increased path degeneracy due to the resampling procedure, which increases the sample variance. The optimal choice for $\varDelta$ balances the opposing influences of the forgetting rate of the model and the collapsing rate of the resampling process due to the divergence between the proposal distribution and the posterior distribution. For the examples in this paper we chose $\varDelta = 20$ as recommended by Cappé and Moulines (2005), which seems to be a reasonable choice for our models.

There are various other techniques to improve on this basic SMC method, including improved resampling schemes (Douc and Cappé 2005; Olsson et al. 2008; Doucet and Johansen 2009; Cappé et al. 2007), and choosing better sampling distributions through lookahead strategies or resample-move procedures (Pitt and Shephard 1999; Lin et al. 2013; Doucet and Johansen 2009), which are not discussed further here. Instead, in the remainder of this paper, we focus on the process of updating the parameter estimates $\hat{ \theta }_{t}$. The remainder of this section describes the options for step 3 of Algorithm 1.

### Batch expectation maximization

Batch Expectation Maximization (BEM) processes the data in batches. Within a batch of size *b*, the parameter estimate stays constant ($\hat{ \theta }_{t}=\hat{ \theta }_{t-1}$) and the update to the sufficient statistic

$$\begin{aligned} \tilde{s}_{t}:=\sum _{i}w_t(X_{1:t}^{(i)}) \cdot (Y_{t-\varDelta }-X_{1:t}^{(i)}(t-\varDelta ))^2, \end{aligned}$$

is collected at each iteration *t*. At the end of the *m*th batch we have $t=mb$, at which time

$$\begin{aligned} \hat{S}^{BEM}_{t}:=\frac{1}{b}\sum _{k=(m-1)b+1}^{mb}\tilde{s}_{k}, \end{aligned}$$

is our approximation of *S*, and $\hat{\sigma }^{2}_{v,t}:=\hat{S}^{BEM}_{t}$.

The batch size determines the convergence behavior of the estimates. For a fixed computational cost, choosing *b* too small will result in noise-dominated estimates and low precision, whereas choosing *b* too large will result in precise but inaccurate estimates due to slow convergence.

### Online expectation maximization

BEM only makes use of the collected evidence at the end of each batch, missing potential early opportunities for improving parameter estimates. OEM addresses this issue by updating the parameter estimate at every iteration. The approximation of *S* at time *t* is a running average of $\{\tilde{s}_{k}\}_{k=\varDelta +1,\ldots ,t}$, weighted by a pre-specified learning schedule. The choice of learning schedule determines how quickly the algorithm “forgets” the earlier parameter estimates. In OEM at time *t*,

4 $$\begin{aligned} \hat{S}^{OEM}_{t}=\gamma _{t} \cdot \tilde{s}_{t} + (1-\gamma _{t}) \cdot \hat{S}^{OEM}_{t-1}, \end{aligned}$$

where $\{\gamma _{t}\}_{t=1,2,\ldots }$ is the chosen learning schedule, typically of the form

5 $$\begin{aligned} \gamma _{t}=t^{-c} \end{aligned}$$

for a fixed choice of $c\in (0.5,1]$ (Cappé 2009). Note that when using lag $\varDelta$, $\gamma _{t}=(t-\varDelta )^{-c}$ for $t \ge \varDelta$. This update rule ensures that at time *t*, $\hat{S}^{OEM}$ is a weighted sum of $\{\tilde{s}_{k}\}_{k=\varDelta +1,\ldots ,t}$ where the term ${\tilde{s}}_k$ has weight

6 $$\begin{aligned} \eta _k^t := \gamma _{k} (1-\gamma _{k+1}) \cdots (1-\gamma _{t-1})(1-\gamma _{t}). \end{aligned}$$

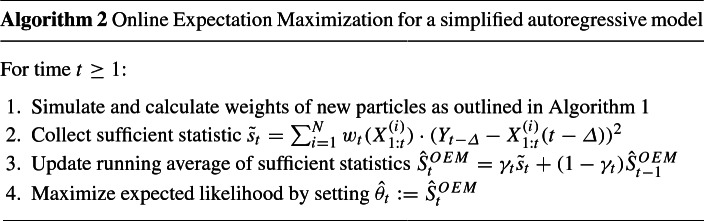


Although this method can outperform BEM as parameters are updated continuously, its performance remains strongly dependent on the parameter *c* determining the learning schedule $\gamma _t$, and a suboptimal choice can reduce performance by orders of magnitude. At one extreme, the estimates will depend strongly only on the most recent data, resulting in noisy parameter estimates and low precision. At the other extreme, the estimates will average out stochastic effects but be severely affected by false initial estimates, resulting in more precise but less accurate estimates. Again, the best choice depends on the model.

A pragmatic approach to the problem of choosing a tuning parameter in OEM takes inspiration from Polyak (1990). In this method, a learning schedule that emphasizes incoming data is used to ensure quick initial convergence, while imprecise estimates are avoided at later iterations by averaging all OEM estimates beyond a threshold $t_0$.

$$\begin{aligned} \hat{\theta }^{AVG}_t = {\left\{ \begin{array}{ll} \hat{\theta }^{OEM}_t &{} \quad \text {for } t < t_0 \\ \frac{1}{t-t_0+1} \sum _{k=t_0}^t \hat{\theta }^{OEM}_k &{} \quad \text {for } t \ge t_0. \end{array}\right. } \end{aligned}$$

Choosing an appropriate threshold $t_0$ can be more straightforward than choosing *c* for $\gamma _t = t^{-c}$, but it still requires the user to have an intuition for how the estimates for each parameter will behave. We will refer to this method as AVG, use $c=0.6$, and set $t_0=50{,}000$ which is half the total iterations for our examples.

### Introspective online expectation maximization

We now introduce IOEM to address the issue of having to pre-specify a learning schedule $\{\gamma _t\}_{t=1,\ldots }$. The algorithm is similar to OEM, but instead of pre-specifying $\gamma _{t}$, we estimate the precision and accuracy in the sufficient statistic updates $\{\tilde{s}_{k}\}_{k=\varDelta +1,\ldots ,t}$ and use these to determine $\gamma _{t+1}$. More precisely, we keep online estimates of a weighted regression on the dependent variables $\{\tilde{s}_{k}\}_{k=\varDelta +1,\ldots ,t}$ where $k-t$ serves as the (shifted) explanatory variable:

7 $$\begin{aligned} \tilde{s}_k = \beta _0 + \beta _1(k-t) + \epsilon _k \end{aligned}$$

where $\epsilon _k\sim N(0,\sigma ^2)$, and data point $(k-t,\tilde{s}_{k})$ has weight (6) as before. This weighted regression results in intercept and slope estimates $\hat{\beta }_0$, $\hat{\beta }_1$ and variance estimates $\hat{\sigma }_0^2$, $\hat{\sigma }_1^2$, where at convergence $\hat{\beta }_0$ is the sought-after estimate and $\hat{\beta }_1\simeq 0$. We do not use standard weighted regression, in which weights are inversely proportional to the variance of the observation, as this assumption is not justified here and would lead to biased estimates of $\hat{\sigma }^2_{0,1}$. Instead we assume that observations share an unknown variance, and we use the weights to modulate the influence of each observation to the regression estimates, to reduce the impact of the bias in earlier observations; see Sect. 7.2 for details.

We next use the regression coefficients to estimate the past iteration where the drift term $|\hat{\beta }_1|(k-t)$ is of the same order as the uncertainty $\hat{\sigma }_0$ in the main estimate $\hat{\beta }_0$:

8 $$\begin{aligned} t - k = \alpha {\hat{\sigma }_0 \over |\hat{\beta }_1| + \hat{\sigma }_1}, \end{aligned}$$

where $\hat{\sigma }_1$ ensures that division by zero does not occur, and $\alpha$ tunes the algorithms’s sensitivity to model misfit due to underlying parameter changes; we use $\alpha =1$ unless stated otherwise. We propose a learning rate $\gamma ^{reg}_{t+1}$ that results in a characteristic forgetting time $1/\gamma ^{reg}_{t+1}$ matching this distance:

9 $$\begin{aligned} \gamma _{t+1}^{reg}=\frac{ |\hat{\beta }_1| + \hat{\sigma }_1 }{ \alpha \hat{\sigma }_0 }. \end{aligned}$$

This choice ensures that a substantial slope estimate $|\hat{\beta }_1|$ indicating that $\hat{\beta }_0$ has low accuracy puts large weight on the incoming statistic, improving accuracy, whereas a large $\hat{\sigma }_0$ reflecting low precision in estimate $\hat{\beta }_0$ results in a small weight, smoothing out successive estimates and improving precision. We impose restrictions on $\gamma _{t+1}$ which keep it between the most extreme valid learning schedules for OEM. Taken together, the update step for $\gamma$ becomes

10 $$\begin{aligned} \gamma _{t+1} = \min \left( (t+1)^{-c}, \max \left( \gamma _{t+1}^{reg}, \gamma _t / (1+\gamma _t) \right) \right) \end{aligned}$$

where $c>0.5$ is chosen to be very close to 0.5 and guarantees convergence. These restrictions ensure that our algorithm satisfies the assumptions of Theorem 1 of Cappé and Moulines (2009), namely that $0< \gamma _t < 1$, $\sum _{t=1}^\infty \gamma _t = \infty$, and $\sum _{t=1}^{\infty } \gamma _t^2 < \infty$. Hence for any model for which *f* and *g* satisfy the assumptions guaranteeing convergence of the standard OEM estimator, the IOEM algorithm is also guaranteed to converge. The precise conditions are detailed in Assumption 1, Assumption 2, and Theorem 1 of Cappé and Moulines (2009).
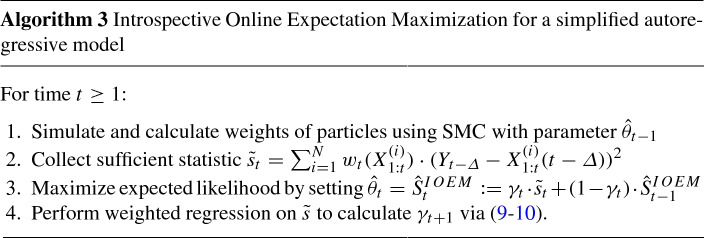


## The IOEM algorithm for the full autoregressive model

The adapting learning schedule $\{\gamma _t\}_{t=1,\ldots }$ sets IOEM apart from OEM. However, the way $\gamma _t$ is calculated in Algorithm 3 only works in the special case that a single sufficient statistic and the single parameter of interest coincide (here, $\hat{\sigma }_{v,t}^2=\hat{S}_t$). In general, the sufficient statistics $\hat{S}$ are mapped to parameter estimates $\hat{\theta }$ by a function $\varLambda$, leading to a more involved setup that we explore here. To this end, we now consider the full noisily-observed autoregressive model AR(1) with master equations as in (1), but now with unknown parameters *a*, $\sigma _w$, and $\sigma _v$. We define four sufficient statistics,

$$\begin{aligned} S_{1,t}&=\mathbb {E}_{X_{1:t} | Y_{1:t}, \theta }\left[ \frac{1}{t-1}\sum _{k=1}^{t-1}X_k^2\right] ,\\ S_{2,t}&=\mathbb {E}_{X_{1:t} | Y_{1:t}, \theta }\left[ \frac{1}{t-1}\sum _{k=1}^{t-1}X_k \cdot X_{k+1}\right] ,\\ S_{3,t}&=\mathbb {E}_{X_{1:t} | Y_{1:t}, \theta }\left[ \frac{1}{t-1}\sum _{k=2}^{t}X_k^2\right] ,\\ S_{4,t}&=\mathbb {E}_{X_{1:t} | Y_{1:t}, \theta }\left[ \frac{1}{t}\sum _{k=1}^{t}(Y_k-X_k)^2\right] . \end{aligned}$$

Then, in BEM and OEM, we update the parameter estimates to

11 $$\begin{aligned} \hat{a}_{t}&=\hat{S}_{2,t}/ \hat{S}_{1,t} , \end{aligned}$$

12 $$\begin{aligned} \hat{\sigma }_{w,t}&=(\hat{S}_{3,t}-(\hat{S}_{2,t})^2/\hat{S}_{1,t})^{1/2} ,\end{aligned}$$

13 $$\begin{aligned} \hat{\sigma }_{v,t}&=(\hat{S}_{4,t})^{1/2} , \end{aligned}$$

where $\hat{S}_t$ is an approximation of $S_t$.

In most cases, as above, the function $\varLambda$ mapping $\hat{S}_{t}$ to $\hat{\theta }_{t}$ is nonlinear, and requires multiple sufficient statistics as input. To avoid bias, we want all sufficient statistics that inform one parameter estimate to share a learning schedule $\{\gamma _{t}\}_{t=1,2,\ldots }$. We therefore estimate an adapting learning schedule for each parameter independently, by performing the regression on the level of the parameter estimates (Algorithm 4), rather than on the level of the sufficient statistics. We will calculate $\hat{S}_t$ as in OEM (4) using our adapting learning schedule instead of a user specified learning schedule. Because the adapting learning schedule is specific to each parameter, we will have multiple estimates of certain summary sufficient statistics. In this case $S_{1,t}$ and $S_{2,t}$ are estimated by $\hat{S}_{1,t}^{a}$ and $\hat{S}_{2,t}^{a}$ for (11) and by $\hat{S}_{1,t}^{\sigma _w}$ and $\hat{S}_{2,t}^{\sigma _w}$ for (12).

Simply regressing on $\hat{\theta }_{1:t}$ with respect to *t* would correspond to regression on $\hat{S}_{1:t}$, not $\tilde{s}_{1:t}$. As $\hat{S}$ is a running average, there is a strong correlation between $\hat{S}_{t-1}$ and $\hat{S}_t$ and hence also a strong dependence between $\hat{\theta }_{t-1}$ and $\hat{\theta }_t$. In order to perform the regression on the parameters we must “unsmooth” $\hat{\theta }_{1:t}$ to create pseudo-independent parameter updates $\tilde{\theta }_t$ (see Algorithm 4). This is accomplished by taking linear combinations,

$$\begin{aligned} \tilde{\theta }_{t}:=\frac{1}{\gamma _{t}} \cdot \hat{\theta }_{t} + \left( 1-\frac{1}{\gamma _{t}}\right) \cdot \hat{\theta }_{t-1}, \end{aligned}$$

where the coefficients are chosen so as to minimize the covariance between successive updates, justifying the term pseudo-independent. The resulting updates correspond with the unsmoothed sufficient statistics updates $\tilde{s}_{t}$ used in Sect. 2.3. See Sect. 7.3 for further details on this step.
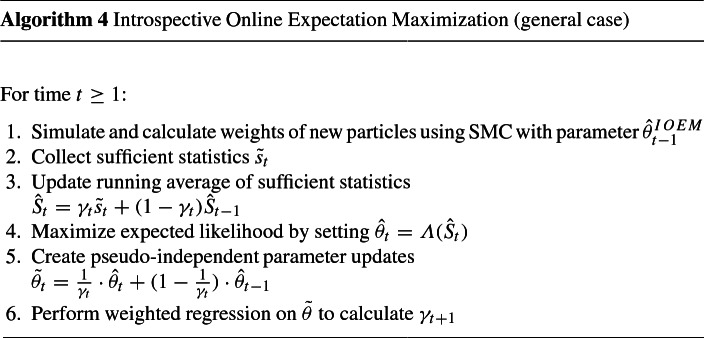


## Simulations

We performed inference on different models using the BEM, OEM and IOEM algorithms as described above. For BEM we used batch sizes from 100 to 10, 000, and for OEM we used learning schedules $\gamma _t=t^{-c}$ with *c* ranging from 0.6 to 0.9. In all cases the bootstrap filter was run with $N=100$ particles, and the algorithm was run from $t=1$ to $t=100{,}000$. For all parameter choices, 100 independent replicates were generated, and we show the distribution of inferred parameter values across these replicates.

### Inference with the simplified IOEM algorithm

**Fig. 1:**
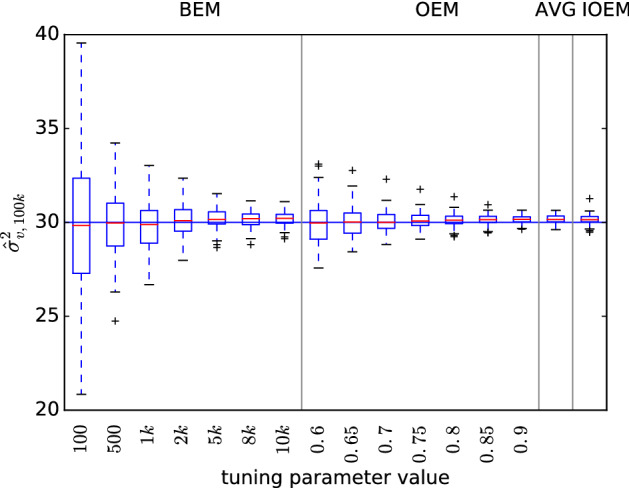
Comparison of EM methods on simplified AR model with known true parameters $a=.95$, $\sigma _w=1$, and unknown true $\sigma _v^2=30$, and initial parameter estimate $\sigma _{v,0}^2=20$. $\hat{\sigma }_{v,100k}^2$ is plotted for 100 replicates, $N=100$

We first applied the simplified IOEM algorithm (Algorithm 3) to the problem of inferring $\sigma _v^2$ in model (1), with all other parameters assumed known, and compared the results with the BEM and OEM algorithms (Fig. 1). The choice of tuning parameter in BEM and OEM makes a significant difference to the precision of the estimate even after 100,000 observations. IOEM was able to recognize that behavior similar to BEM with $b=10{,}000$ or OEM with $c=0.9$ was optimal. The accuracy and precision of IOEM are comparable with those of the post-OEM averaging technique (AVG) with parameters $c=0.6$ and $t_0=50{,}000$.

### Inference with the complete IOEM algorithm

**Fig. 2:**
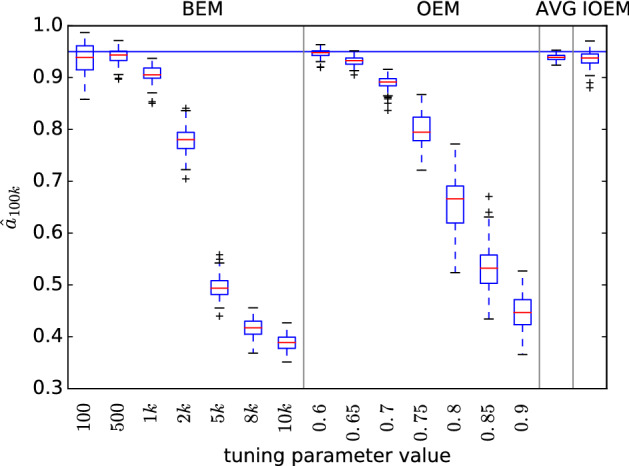
Comparison of EM methods on full autoregressive model with unknown true parameters $a=0.95$, $\sigma _{w}=1$, $\sigma _{v}=5.5$ and initial parameters $a_{0}=0.8$, $\sigma _{w,0}=3$, $\sigma _{v,0}=1$. $\hat{a}_t$ at $t=100{,}000$ is plotted for 100 replicates, $N=100$

We next treated all four parameters of the AR(1) model (1) as unknown, and inferred them using the full IOEM algorithm (Algorithm 4). Estimates for the *a* parameter under different EM methods are presented in Fig. 2; for the other parameter inferences see Sect. 7.5, Fig. 6.

In the AR(1) model, IOEM outperforms most other EM methods when estimating the *a* parameter, while AVG for the chosen parameter settings ($c=0.6$, $t_0=50{,}000$) provides slightly more precise estimates at similar accuracy. It is worth noting that in this case, OEM with $c=0.6$ substantially outperforms OEM with $c=0.9$, in contrast to the results shown in Fig. 1. This is a result of the bad initial estimates. OEM with $c=0.6$ forgets the earlier simulations much faster than OEM with $c=0.9$ and hence is able to move its estimates of *a*, $\sigma _w$, and $\sigma _v$ much more quickly. Here IOEM recognizes that it should have similar behavior to OEM with $c=0.6$, whereas in the inference displayed in Fig. 1 IOEM chose behavior similar to OEM with $c=0.9$. IOEM can indeed adapt to the model.

### Inference of multiple parameters

Next we investigated a model with a larger number of parameters and varying accuracy of initial parameter estimates. One of the advantages of the IOEM algorithm over OEM is its ability to adapt to each parameter independently. To highlight this, we applied IOEM to a simple 2-dimensional autoregressive model. For this model we consider the sequences $\{Y^A,Y^B\}_{1:t}$ as observed, while $\{X^A,X^B\}_{1:t}$ are unobserved, where

14 $$\begin{aligned} X^{A}_{t}&=a^{A} X^{A}_{t-1}+ \sigma ^{A}_{w} W^{A}_{t},&X^{B}_{t}&=a^{B} X^{B}_{t-1}+ \sigma ^{B}_{w} W^{B}_{t}, \nonumber \\ Y^{A}_{t}&=X^{A}_{t} + \sigma _{v} V^{A}_{t},&Y^{B}_{t}&=X^{B}_{t} + \sigma _{v} V^{B}_{t}. \end{aligned}$$

Note that $Y^A$ and $Y^B$ are uncoupled, and that their master equation have independent parameters except for a shared parameter $\sigma _v$. By giving component *A* good initial estimates and *B* bad initial estimates, we can see how the different EM methods cope with a combination of accurate and inaccurate initializations. IOEM is able to identify the set with good initial estimates ($a^A,\sigma ^{A}_{w}$) and quickly start smoothing out noise. To IOEM, the other parameters appear to not have converged ($\sigma ^{B}_{w}$ and $\sigma _{v}$ because they are at the wrong value, $a^B$ because it will be changing to compensate for $\sigma ^{B}_{w}$ and $\sigma _{v}$).

**Fig. 3:**
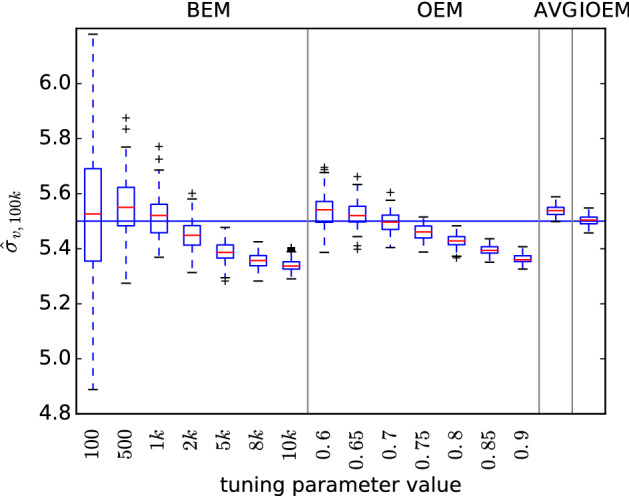
Comparison of EM methods on 2-dimensional autoregressive model with true parameters $a^A=0.95$, $\sigma ^{A}_{w}=1$, $\sigma _{v}=5.5$, $a^B=0.95$, $\sigma ^{B}_{w}=1$ and initial parameters $a^{A}_{0}=0.95$, $\sigma ^{A}_{w,0}=1$, $\sigma _{v,0}=3$, $a^{B}_{0}=0.95$, $\sigma ^{B}_{w,0}=3$. $\hat{\sigma }_{v,t}$ at $t=100{,}000$ is plotted for 100 replicates, $N=100$

Figure 3 shows the inference of $\sigma _v$, which due to its dependence on components A and B, suffers the most from a blanket choice of tuning parameter in BEM or OEM. OEM with $c=0.6$ and OEM with $c=0.9$ both suffer in this model as they are both well suited to parameter estimation in one of the components, but not the other. AVG provides precise but biased estimates in this case, because of its reliance on a fast-forgetting initial OEM stage which again is suited to only one of the model components. IOEM on the other hand is able to capture the best of both worlds, striving for precision in component A and initially foregoing precision in favour of accuracy in component B.

The inference of the other parameters and comparisons with a different choice of AVG threshold are shown in Sect. 7.5, Figs. 7, 8, 9, 10.

### Inference of parameters of a stochastic volatility model

The previous sections have demonstrated IOEM is comparable to choosing the optimal tuning parameter in OEM or BEM in certain models. However, the models shown have all been based on the noisily observed autoregressive model, which is a linear Gaussian case where in practice analytic techniques would be preferred over SAEM. We now examine the behaviour of these algorithms when inferring the parameters of a non-linear stochastic volatility model defined by transition and emission densities

15 $$\begin{aligned} f(x_t|x_{t-1})&= (2 \pi \sigma ^2)^{-1/2} \exp \Big \{-\frac{(x_t - \phi x_{t-1})^2}{2 \sigma ^2}\Big \}, \end{aligned}$$

16 $$\begin{aligned} g(y_t|x_t)&= (2 \pi \beta ^2 e^{x_t} )^{-1/2} \exp \Big \{-\frac{ 1 }{ 2 \beta ^2 e^{x_t} } y^2_t \Big \}. \end{aligned}$$

We define four summary sufficient statistics,

$$\begin{aligned} S_{1,t}&=\mathbb {E}_{X_{1:t} | Y_{1:t}, \theta }\left[ \frac{1}{t-1}\sum _{k=1}^{t-1}X_k \cdot X_{k+1}\right] ,\\ S_{2,t}&=\mathbb {E}_{X_{1:t} | Y_{1:t}, \theta }\left[ \frac{1}{t-1}\sum _{k=1}^{t-1}X_k^2\right] ,\\ S_{3,t}&=\mathbb {E}_{X_{1:t} | Y_{1:t}, \theta }\left[ \frac{1}{t-1}\sum _{k=2}^{t}X_k^2\right] ,\\ S_{4,t}&=\mathbb {E}_{X_{1:t} | Y_{1:t}, \theta }\left[ \frac{1}{t}\sum _{k=1}^{t} e^{-X_k} \cdot Y_k^2 \right] . \end{aligned}$$

Then the set of parameters that maximises the likelihood at step *t* are

17 $$\begin{aligned} \hat{\phi }_{t}&=\hat{S}_{1,t}/ \hat{S}_{2,t} , \end{aligned}$$

18 $$\begin{aligned} \hat{\sigma }_{t}&=(\hat{S}_{3,t}-(\hat{S}_{1,t})^2/ \hat{S}_{2,t})^{1/2} , \end{aligned}$$

19 $$\begin{aligned} \hat{\beta }_{t}&=(\hat{S}_{4,t})^{1/2} , \end{aligned}$$

**Fig. 4:**
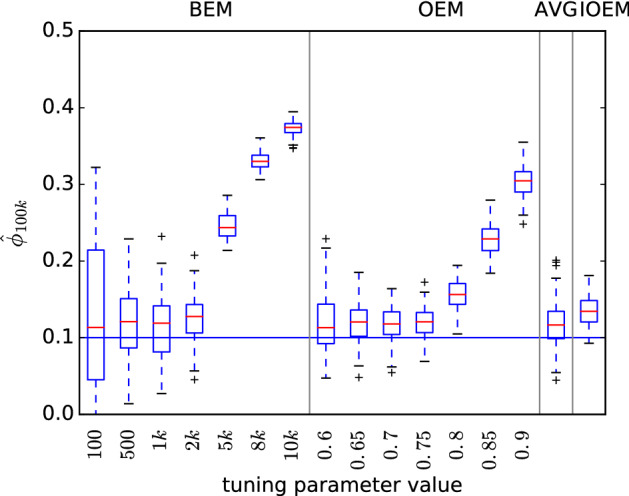
Estimates of $\phi$ in stochastic volatility model

Again IOEM results in similar estimates to the optimal BEM/OEM and the online averaging technique with a well-chosen threshold (see Fig. 4 and Sect. 7.5, Fig. 11).

## Application to financial time series

**Fig. 5:**
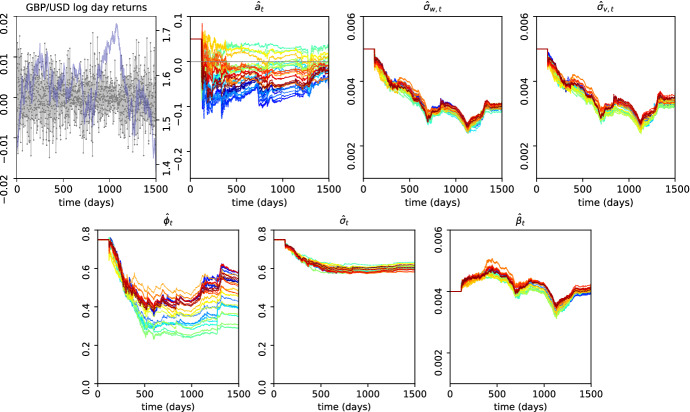
Running estimates of parameters of model (1) (top) and model (16) (bottom) on time series of daily GBP/USD log returns (top left panel) based on 24 different hourly offsets (colours) using IOEM ($N=5000$). We used $\alpha =2$ to reduce the impact of underlying parameter changes; for results with $\alpha =1$ see Figs. 15 and 16 (color figure online)

**Fig. 6:**
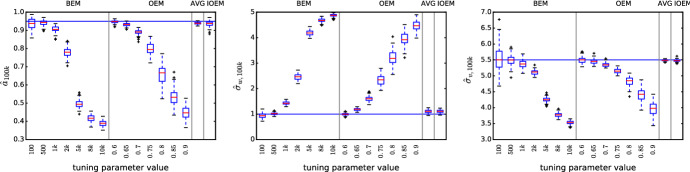
Comparison of EM methods on full autoregressive model with unknown true parameters $a=0.95$, $\sigma _{w}=1$, $\sigma _{v}=5.5$ and initial parameters $a_{0}=0.8$, $\sigma _{w,0}=3$, $\sigma _{v,0}=1$. Parameter estimates at $t=100{,}000$ are plotted for 100 replicates, $N=100$

**Fig. 7:**
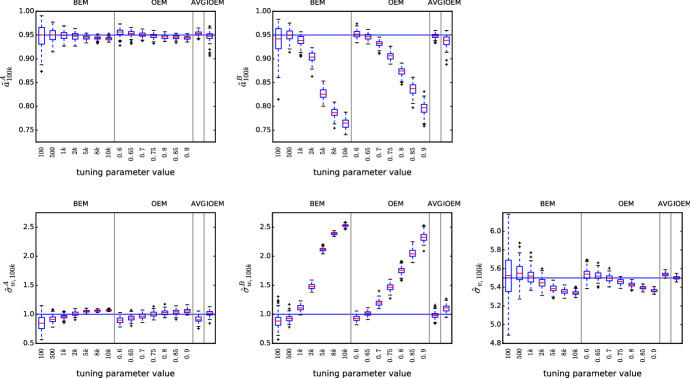
Comparison of EM methods on 2-dimensional autoregressive model with true parameters $a^A=0.95$, $\sigma ^{A}_{w}=1$, $\sigma _{v}=5.5$, $a^B=0.95$, $\sigma ^{B}_{w}=1$ and initial parameters $a^{A}_{0}=0.95$, $\sigma ^{A}_{w,0}=1$, $\sigma _{v,0}=3$, $a^{B}_{0}=0.95$, $\sigma ^{B}_{w,0}=3$. Parameter estimates at $t=100{,}000$ are plotted for 100 replicates, $N=100$

**Fig. 8:**
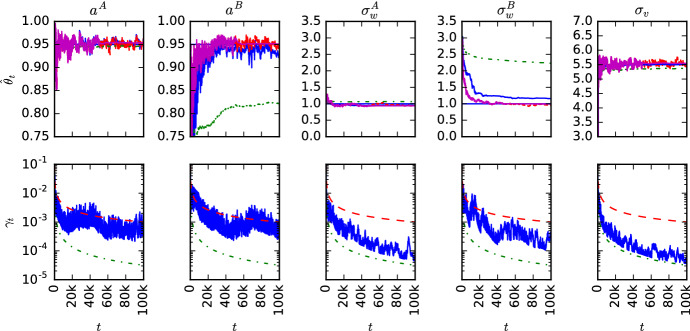
Parameter-specific convergence in the 2-dimensional autoregressive model over 100,000 observations. Each column displays information for a single parameter. The top row shows the sequence of parameter estimates for three EM methods. The bottom row shows the learning schedule $\gamma _t$ for the three EM methods. Blue solid line: IOEM; red dashed line: OEM with $c=0.6$; green dash-dot line: OEM with $c=0.9$; magenta solid line: averaged OEM technique with a threshold $t_0=50{,}000$ (color figure online)

We next applied our approach to daily log returns for US dollar to UK pound exchange rates, obtained from oanda.com. Between 18/05/2010 to 2/3/2016, roughly the period between the 2010 flash crash and the Brexit referendum, rates were fairly stable and might be described by an ARMA(1,1) model equivalent to (1). To assess confidence in estimates, we independently inferred model parameters 24 times from day-on-day log returns measured at every full hour (Fig. 5). We note that these time series are not fully comparable due to intraday seasonalities (Cornett et al. 1995), an effect that may be expected to increase the observed variation between the 24 time series, which would lead to conservative confidence estimates. Results suggest a weak negative correlation of successive daily log returns ($a<0$), which is supported by a direct fit of an ARMA(1,1) model to the data (Fig. 12). Although the ARMA(1,1) model assumes fixed parameters and in particular constant volatility, running inferences strongly indicate volatility variations (Figs. 5 and 13), suggesting model (16) might be appropriate. Inferred values of $\phi$ indicate substantial day-to-day inertia in volatility. Running estimates of parameters are fairly constant in time, although those for $\beta$ show that the model has difficulty tracking the two sudden drops in volatility that occurred in this period, indicating model misfit.

## Conclusion

**Fig. 9:**
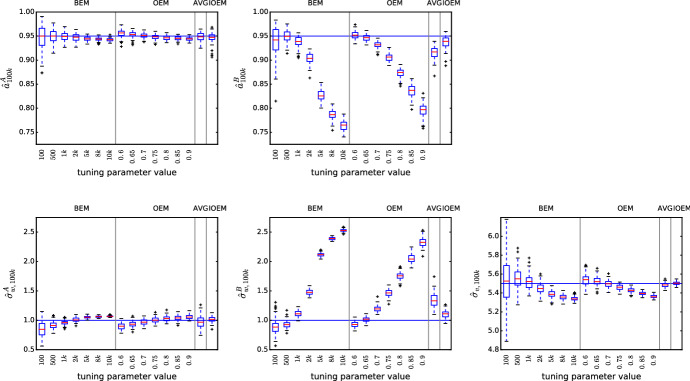
Comparison of EM methods on 2-dimensional autoregressive model with true parameters $a^A=0.95$, $\sigma ^{A}_{w}=1$, $\sigma _{v}=5.5$, $a^B=0.95$, $\sigma ^{B}_{w}=1$ and initial parameters $a^{A}_{0}=0.95$, $\sigma ^{A}_{w,0}=1$, $\sigma _{v,0}=3$, $a^{B}_{0}=0.95$, $\sigma ^{B}_{w,0}=3$. Parameter estimates at $t=100{,}000$ are plotted for 100 replicates, $N=100$

**Fig. 10:**
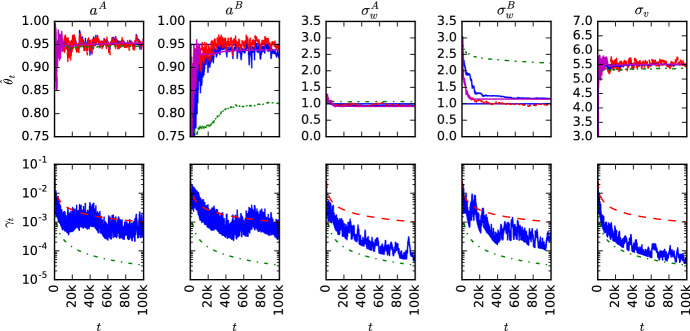
Parameter-specific convergence in the 2-dimensional autoregressive model over 100,000 observations. Each column displays information for a single parameter. The top row shows the sequence of parameter estimates for four EM methods. The bottom row shows the learning schedule $\gamma _t$ for the three EM methods. Blue solid line: IOEM; red dashed line: OEM with $c=0.6$; green dash-dot line: OEM with $c=0.9$; magenta solid line: averaged OEM technique with a threshold $t_0=10{,}000$ (color figure online)

**Fig. 11:**
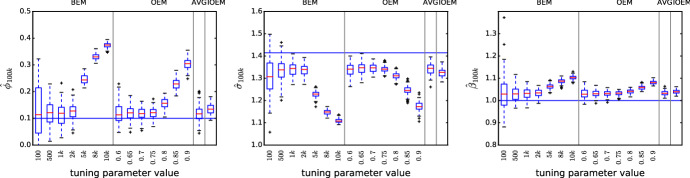
Comparison of EM methods on stochastic volatility model with unknown true parameters $\phi =0.1$, $\sigma =\sqrt{2}$, $\beta =1$ and initial parameters $\phi _{0}=0.5$, $\sigma _{0}=1$, $\beta _{0}=\sqrt{2}$. Parameter estimates at $t=100{,}000$ are plotted for 100 replicates, $N=100$

**Fig. 12:**
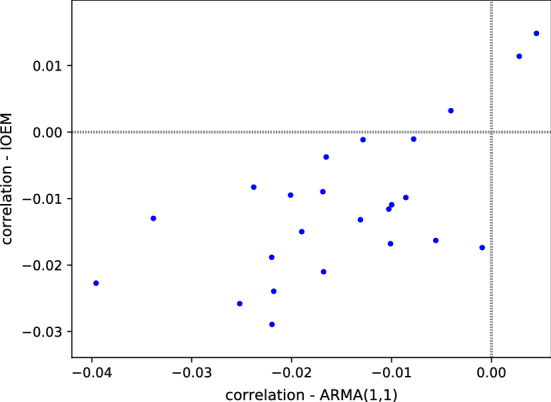
Implied day-to-day correlation of log returns $\rho _{IOEM}$, $\rho _{ARMA(1,1)}$ for the 24 daily time series, calculated as $\rho _{IOEM} = {a / (1 + (1-a^2) {\sigma _v^2 /\sigma _w^2} )}$ and $\rho _{ARMA(1,1)} = {1 + 2 \phi \theta + \theta ^2 / (\phi + \theta )(1 + \phi \theta )}$, where the AR coefficient $\phi$, *a* governs the asymptotic falloff in correlation in the two parameterizations, and $\theta$ is the coefficient of the moving average component. Results indicate a non-significant trend for a negative day-to-day correlation of log returns across the period studied. Inferred values of $\rho$ across 24 different hourly offsets are themselves correlated but not identical between the two models, as expected as IOEM emphasizes recent observations over earlier ones, in contrast to the global optimization used for fitting the ARMA(1,1) model

**Fig. 13:**
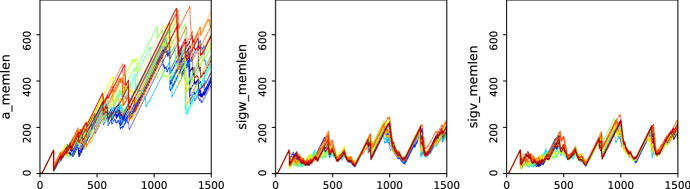
Memory length ($\gamma _t^{-1})$ for the three parameters of model (1) for the 24 daily time series. The data does not appear to support a stationary model, particularly for $\sigma _w$ and $\sigma _v$, as can be seen from the collapse of the memory length $\gamma _{t}^{-1}$ particularly around $t=650$ and $t=1150$, coinciding with periods of low volatility

Stochastic Approximation EM is a general and effective technique for estimating parameters in the context of SMC. However, convergence can be slow, and improving convergence speed is of particular interest in this setting. We have shown that IOEM produces accurate and precise parameter estimates when applied to continuous state-space models. Across models, and across varying levels of accuracy of the initial estimates, the efficiency of IOEM matches that of BEM/OEM with the optimal choice of tuning parameter. The AVG procedure also shows good behaviour, but like BEM/OEM it has tuning parameters, and when these are chosen suboptimally performance is not as good as IOEM (Figs. 9 and 10). BEM/OEM/AVG all make use of a single learning schedule $\{\gamma _{t}\}$, and for more complex models a single learning schedule generally cannot achieve optimal convergence rates for all parameters, as we have shown for the 2-dimensional AR example. In addition, AVG works by post-hoc averaging of noisy estimates, and since the inferences depend on the noisy estimates themselves, this implicitly relies on the model being sufficiently linear around the true parameter value. We expect IOEM to be more resilient to strong nonlinearities than AVG, but we have not explored this idea further here.

IOEM finds parameter-specific learning schedules, resulting in better performance than standard methods with a single learning rate parameter are able to achieve. IOEM can be applied with minimal prior knowledge of the model’s behavior, and requires no user supervision, while retaining the convergence guarantees of BEM/OEM, therefore providing an efficient, practical approach to parameter estimation in SMC methods. While not the focus of this paper, application to a financial time series suggests that IOEM may be useful in informally assessing model fit; it would be interesting to investigate whether this could be made rigorous.

## Figures and Tables

**Fig. 14:**
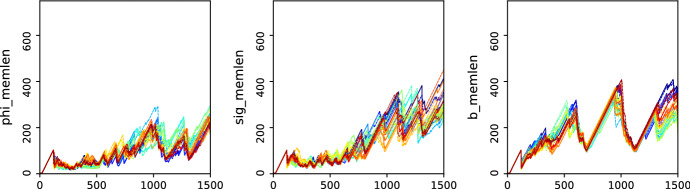
Memory length ($\gamma _t^{-1})$ for the three parameters of model (16) for the 24 daily time series. The inferred parameters (see Fig. 5) appear to support a stationary model, although the collapse in memory length for parameter $\beta$ due to apparent drift indicates the model’s difficulty in tracking sudden changes in volatility around $t=650$ and $t=1150$

**Fig. 15:**
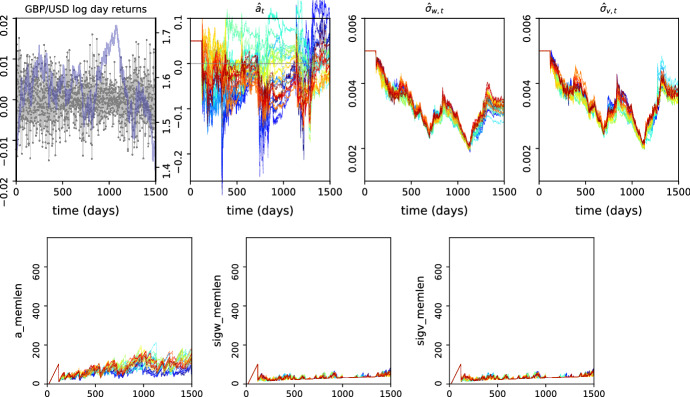
Running estimates of parameters of model (1) on daily GBP/USD log returns, and memory length, inferred using $\alpha =1$

**Fig. 16:**
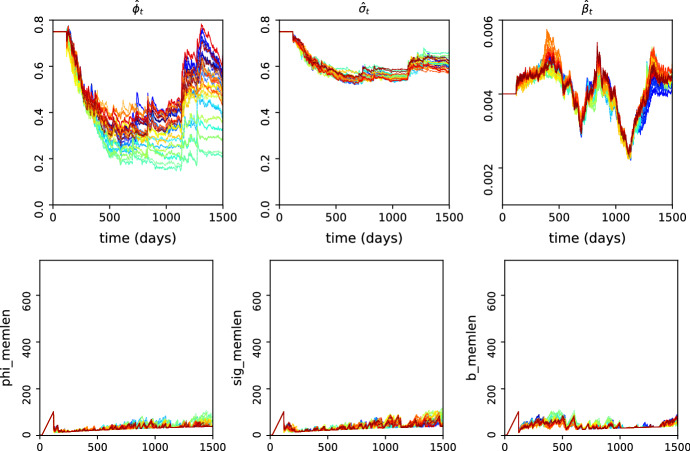
Running estimates of parameters of model (16) on daily GBP/USD log returns, and memory length, inferred using $\alpha =1$

## Appendix

### Fixed-lag technique

Our fixed-lag technique is slightly different than that proposed in the literature (Cappé and Moulines 2005; Olsson et al. 2008). Compared to the existing approach it uses less intermediate storage. Recall that the approximation we aim to evaluate is

$$\begin{aligned} \hat{S}_t = \sum _i w_t(X_{1:t}^{(i)}) \cdot \sum _{u=1}^t s_u( X_{1:t}^{(i)}(u), Y(u) ), \end{aligned}$$

where the sufficient statistic is written explicitly as a sum over the path traced out by the particle $X_{1:t}^{(i)}$. The drawback is that for $u \ll t$ the paths will have collapsed due to resampling, increasing the variance for those contributions to *S*. The solution proposed in Cappé and Moulines (2005) is to use instead the approximation

$$\begin{aligned} \hat{S}_t&\approx \sum _i \bigg ( \sum _{u=1}^{t-\varDelta } w_{u+\varDelta }(X_{1:u+\varDelta }^{(i)}) s_u( X_{1:u+\varDelta }^{(i)}(u), Y(u) ) \\&\qquad \qquad +\, w_t(X_{1:t}^{(i)}) \sum _{u=t-\varDelta +1}^{t} s_u( X_{1:t}^{(i)}(u), Y(u) ) \bigg ). \end{aligned}$$

This requires storing the quantities

$$\begin{aligned} \{ s_u(X_{1:u+\varDelta }^{(i)}(u)), Y(u) \}_{u=t-\varDelta ,\ldots ,t} \end{aligned}$$

for each sufficient statistic and each particle. This storage can be expensive if large numbers of sufficient statistics are tracked. Instead, at iteration *t* we use the approximation

$$\begin{aligned} \hat{S}_{t} \approx \sum _{u=1}^{t-\varDelta } \sum _i w_{u+\varDelta }(X^{(i)}_{1:u+\varDelta }) s_u(X^{(i)}_{1:u+\varDelta }(u),Y(u)). \end{aligned}$$

By disregarding terms involving $s_u$ for $u>t-\varDelta$ and switching the summation in this way, we can now update $\hat{S}$ at each iteration by adding the contribution of the current particles to a single summary statistic at a distance $\varDelta$, without requiring per-particle storage other than each particle’s recent history.

### Weighted regression

The term “weighted regression” usually refers to regression where the errors are independent and normally distributed with zero mean and known variance (up to a multiplicative constant), and the data is weighted inversely proportionally to its variance. In our case, the data is assumed to drift, contributing an additional, non-independent term to the error. Weights are used to only focus on recent data where the drift contributes an error of the same order of magnitude as the normally distributed noise, while discounting the impact of data points further away. In this setup we are interested both in estimating the regression coefficients, and the error in these estimates.

Perry Kaufman’s adaptive moving average (AMA) (Kaufman 1995) is a similar averaging technique which reacts to the trends and volatility (jointly referred to as the behavior) of the sequence. The difference lies in the measure of the behavior. AMA relies on a user specified window length *n*. The *n* most recent data points are used to measure the behavior. This would be equivalent to using equally-weighted linear regression over the last *n* points. By using weighted regression, the contribution of points to the behavior measures is also influenced by the previously observed behavior. For example, a sharp trend will effectively employ a smaller *n* value as we have lost interest in the behavior before that trend.

Let *X* be the $2\times n$ matrix consisting of a column of 1s and a column with the dependent variable, let *y* be the vector of observations, let $\beta$ be the two coefficients, and $\epsilon$ the vector of errors, with $\epsilon _k \sim N(0,\sigma ^2)$. Finally let *w* be a vector of weights. We estimate $\beta$ by minimizing

$$\begin{aligned} s^2&= (X_w\beta - y_w)^\top (X_w\beta - y_w), \end{aligned}$$

where $X_w$ and $y_w$ are defined as

$$\begin{aligned} X_w:=\begin{bmatrix} w_1&w_1 \cdot (-n+1) \\ \vdots&\vdots \\ w_n&w_n \cdot 0 \end{bmatrix};\qquad y_w:=\begin{bmatrix} w_1 \cdot y_1 \\ \vdots \\ w_n \cdot y_n \end{bmatrix}. \end{aligned}$$

Setting the derivative $\partial s^2/\partial \beta = 2(X_w\beta - y_w)^\top X_w$ to zero and solving for $\beta$ results in weighted regression estimator $\hat{\beta } = (X^\top _w X_w)^{-1}X^\top _w y_w$, or explicitly

$$\begin{aligned} \hat{\beta }_1&= \frac{ \left( \sum w_k^2 x_{2k} y_k\right) - \left( \sum w_k^2 x_{2k} \right) \left( \sum w _k^2 y_k\right) }{ \left( \sum w_k^2 x_{2k}^2\right) - \left( \sum w_k^2 x_{2k}\right) ^2 }, \\ \hat{\beta }_0&= \frac{ \left( \sum w_k^2 x_{2k}^2 \right) \left( \sum w_k^2 y_k\right) - \left( \sum w_k^2 x_{2k} y_k \right) \left( \sum w_k^2 x_{2k} \right) }{ \left( \sum w_k^2 x_{2k}^2\right) - \left( \sum w_k^2 x_{2k}\right) ^2 }. \end{aligned}$$

From this expression we can see that $\hat{\beta }$ can be updated in an online manner as *k* increases simply by updating the above summations. The variance in $\hat{\beta }$ can be estimated as follows:

$$\begin{aligned} {{\,\mathrm{var}\,}}\hat{\beta }&= {{\,\mathrm{var}\,}}(X^\top _w X_w)^{-1} X^\top _w y_w \\&= E \left[ (X^\top _w X_w)^{-1} X^\top _w \epsilon _w \epsilon _w^\top X_w (X^\top _w X_w)^{-1} \right] \\&= (X^\top _w X_w)^{-1} X^\top _w {{\,\mathrm{diag}\,}}( w_k^2 \sigma ^2 ) X_w (X^\top _w X_w)^{-1}. \end{aligned}$$

If $w_k^2=1$ this simplifies to the usual ${{\,\mathrm{var}\,}}\hat{\beta } = \sigma ^2 (X^\top X)^{-1}$. Writing out the expression for ${{\,\mathrm{var}\,}}\hat{\beta }$ explicitly shows that it is again possible to find online updates for the relevant terms.

### Pseudo-independent parameter updates

In order to perform our regression on the level of the parameters, we need to map from $\tilde{s}^{(t)}$ to $\hat{S}^{(t)}$ and then to $\hat{\theta }^{(t)}$. We do not wish to regress on $\hat{\theta }^{(1:t)}$, as $\hat{\theta }^{(t-1)}$ and $\hat{\theta }^{(t)}$ are highly correlated. Instead we want a sequence defined in the parameter space where the correlations resemble those in $\tilde{s}^{(1:t)}$. We define this sequence as

$$\begin{aligned} \tilde{\theta }_{t}:=\frac{1}{\gamma _{t}}\hat{\theta }_{t}+\left( \frac{\gamma _{t}-1}{\gamma _{t}}\right) \hat{\theta }_{t-1}. \end{aligned}$$

Here we show that $\tilde{\theta }_{i}$ and $\tilde{\theta }_{j}$ are uncorrelated for all $i\ne j$, under the assumption that $\tilde{s}_{i}$ and $\tilde{s}_{j}$ are uncorrelated ($i\ne j$). Define $\{\eta _k^t \}_{k=0,\ldots ,t}$ to be the sequence that satisfies $\hat{S}_t = \sum _{k=0}^t \eta _k^t \tilde{s}_k$ and $\sum _{k=0}^t \eta _k^t = 1$. Note that $\eta _t^t=\gamma _t$, $\eta _{t-1}^t=\gamma _{t-1} (1-\gamma _t)$, and so on. Now,

20 $$\begin{aligned} {{\,\mathrm{cov}\,}}(\tilde{\theta }_{i}, \tilde{\theta }_{j}) =\,&{{\,\mathrm{cov}\,}}\biggl ( \frac{1}{ \gamma _{i} } \hat{\theta }_{i} + \frac{ \gamma _{i}-1 }{ \gamma _{i} } \hat{\theta }_{i-1} , \frac{1}{ \gamma _{j} } \hat{\theta }_{j} + \frac{ \gamma _{j}-1 }{ \gamma _{j} } \hat{\theta }_{j-1} \biggr ) \nonumber \\ =\,&\frac{1}{\gamma _i \gamma _j} {{\,\mathrm{cov}\,}}( \hat{\theta }_i,\hat{\theta }_j ) \nonumber \\&+ \frac{1}{\gamma _j} \left( 1-\frac{1}{\gamma _i}\right) {{\,\mathrm{cov}\,}}( \hat{\theta }_{i-1}, \hat{\theta }_j ) \nonumber \\&+ \frac{1}{\gamma _i} \left( 1-\frac{1}{\gamma _j}\right) {{\,\mathrm{cov}\,}}( \hat{\theta }_i, \hat{\theta }_{j-1} ) \nonumber \\&+ \left( 1-\frac{1}{\gamma _i}\right) \left( 1-\frac{1}{\gamma _j}\right) {{\,\mathrm{cov}\,}}( \hat{\theta }_{i-1}, \hat{\theta }_{j-1} ). \end{aligned}$$

Writing $\hat{\theta }_i=f_0+f_1\sum _{k=0}^i \eta _k^i \tilde{s}_k$ and recalling that

$$\begin{aligned} {{\,\mathrm{cov}\,}}(\tilde{s}_i,\tilde{s}_j) = {\left\{ \begin{array}{ll} 0, &{}\quad \text {if } i \ne j \\ \sigma _i^2, &{} \quad \text {if } i=j, \end{array}\right. } \end{aligned}$$

it follows that

$$\begin{aligned} {{\,\mathrm{cov}\,}}(\hat{\theta }_i,\hat{\theta }_j)&= {{\,\mathrm{cov}\,}}\Big ( f_1 \sum _{k=0}^i \eta _k^i \tilde{s}_k,f_1 \sum _{k=0}^j \eta _k^j \tilde{s}_k \Big ) \nonumber \\&= \sum _{k=0}^i f_1^2 \eta _k^i \eta _k^j \sigma _i^2, \end{aligned}$$

for $i<j$. Substituting into the four terms of (20) yields

$$\begin{aligned} {{\,\mathrm{cov}\,}}(\tilde{\theta }_i,\tilde{\theta }_j) =\,&\frac{1}{\gamma _i \gamma _j} \sum _{k=0}^i f_1^2 \eta _k^i \eta _k^j \sigma _k^2 \\&+ \frac{1}{\gamma _j } \left( \frac{\gamma _i-1}{\gamma _i} \right) \sum _{k=0}^{i-1} f_1^2 \eta _k^{i-1} \eta _k^j \sigma _k^2 \\&+ \frac{1}{\gamma _i } \left( \frac{\gamma _j-1}{\gamma _j} \right) \sum _{k=0}^{i} f_1^2 \eta _k^{i} \eta _k^{j-1} \sigma _k^2 \\&+\left( \frac{\gamma _i-1}{\gamma _i } \right) \left( \frac{\gamma _j-1}{\gamma _j} \right) \sum _{k=0}^{i-1} f_1^2 \eta _k^{i-1} \eta _k^{j-1} \sigma _k^2. \end{aligned}$$

If we define

$$\begin{aligned} a&:= f_1^2 \eta _i^i \eta _i^{j-1} \sigma _i^2,\\ b&:= \sum _{k=0}^{i-1} f_1^2 \eta _k^{i-1} \eta _k^{j-1} \sigma _k^2, \end{aligned}$$

and note that

$$\begin{aligned} \eta _k^j=(1-\gamma _j) \eta _k^{j-1} \quad \text {for all } k<j, \end{aligned}$$

then

$$\begin{aligned} {{\,\mathrm{cov}\,}}(\tilde{\theta }_i,\tilde{\theta }_j)&= \frac{1}{\gamma _i \gamma _j} (1-\gamma _j) a + \frac{1}{\gamma _i \gamma _j} (1-\gamma _i) (1-\gamma _j) b \nonumber \\&\quad +\, \frac{1}{\gamma _j } \left( \frac{\gamma _i-1}{\gamma _i} \right) (1-\gamma _j) b \nonumber \\&\quad +\, \frac{1}{\gamma _i } \left( \frac{\gamma _j-1}{\gamma _j} \right) a + \frac{1}{\gamma _i } \left( \frac{\gamma _j-1}{\gamma _j} \right) (1-\gamma _i) b \nonumber \\&\quad +\, \left( \frac{\gamma _i-1}{\gamma _i } \right) \left( \frac{\gamma _j-1}{\gamma _j} \right) b \nonumber \\&= 0. \end{aligned}$$

Hence, if $\tilde{s}_{i}$ and $\tilde{s}_{j}$ are independent for all $i\ne j$, then $\tilde{\theta }_i$ and $\tilde{\theta }_j$ are uncorrelated ($i \ne j$), justifying the term “pseudo-independent updates” for $\tilde{\theta }_i$.

### Notation reference

See Table 1.

**Table 1:** Notation used in this paper

Notation	Meaning	Associated methods
$\theta$	True parameter	All
$\hat{\theta }_t$	Parameter estimate at time *t*	All
$\tilde{\theta }_t$	Pseudo-independent parameter update	IOEM
$\tilde{s}_t$	Sufficient statistic update at time *t*	All
$\hat{S}_t$	Summary sufficient statistic from averaging $\tilde{s}$	All
*N*	Number of particles	All
$\varDelta$	Lag of fixed-lag technique	All
$\hat{\beta }_0$	Regression intercept ML estimate	IOEM
$\hat{\beta }_1$	Regression slope ML estimate	IOEM
$\hat{\sigma }_0^2$	Variance of regression intercept ML estimate	IOEM
$\hat{\sigma }_1^2$	Variance of regression slope ML estimate	IOEM

### Figures

See Figs. 6, 7, 8, 9, 10, 11, 12, 13, 14, 15 and 16.
